# Enhancing the psychological well-being and sleep quality of healthcare providers with a multimodal psychological support program: a randomized controlled trial

**DOI:** 10.3389/fpubh.2024.1455174

**Published:** 2024-12-24

**Authors:** Qi Gao, Yuanyuan Yao, Ruiyu Wang, Xinyue Zhang, Lisa M. Gudenkauf, Guangxin Xu, Samantha Harrison, Leilei Zheng, Jingping Wang, Guanqing Chen, Bin Zheng, Haobo Ma, Min Yan

**Affiliations:** ^1^Department of Anesthesiology, The Second Affiliated Hospital, Zhejiang University, Hangzhou, China; ^2^Medical Psychological Center, The Second Xiangya Hospital, Central South University, Changsha, China; ^3^Department of Health Outcomes and Behavior, Moffitt Cancer Center, Tampa, FL, United States; ^4^Department of Anesthesia, Critical Care and Pain Medicine, Beth Israel Deaconess Medical Center, Harvard Medical School, Boston, MA, United States; ^5^Department of Psychiatry, Second Affiliated Hospital, Zhejiang University School of Medicine and Liangzhu Laboratory, Zhejiang University School of Medicine, Hangzhou, China; ^6^Department of Anesthesia, Critical Care and Pain Management, Massachusetts General Hospital, Boston, MA, United States; ^7^Department of Surgery, University of Alberta, Edmonton, AB, Canada

**Keywords:** COVID-19, multimodal psychological support, healthcare providers, psychological health, public health

## Abstract

**Background:**

The COVID-19 pandemic significantly challenged the global healthcare system, especially frontline healthcare professionals, such as those working in intensive care units (ICUs). In late 2022, a sudden increase in COVID-19 cases in China led to a large number of ICU admissions, requiring new ICU staff (non-ICU professionals to work in ICUs), exacerbating their stress. This study aimed to develop an effective stress management strategy for new ICU professionals, focusing on reducing the detrimental effects of stress on their psychological state. We hypothesized that the online multimodal psychological support (MPS) program might improve the psychological well-being and sleep quality of the participants.

**Methods:**

This single-center, single-blind randomized controlled trial included new ICU staff during the COVID-19 pandemic. Participants were randomly assigned to either an intervention (online psychological support, MPS) or a control (routine wellness care, RWC) group for 28 days, and assessments were conducted before intervention (baseline), after intervention, and at the 1-month follow-up. The intervention included music therapy, sleep hygiene education, psychoeducation, and relaxation training, tailored to address common psychiatric issues experienced by healthcare professionals during the pandemic. The primary outcome was a DASS-21 score 28 days after the end of the intervention.

**Results:**

One hundred and one professionals eventually participated in the study, 47 in the MPS group and 54 in the RWC group. No significant differences were observed in the overall psychological well-being immediately after the end of the intervention. However, the MPS group showed improved sleep and sustained lower stress levels, anxiety, and depression scores at the 1-month follow-up, significantly improving the severity of insomnia (marginal mean difference −2.028; SE 1.00; *p* = 0.044).

**Conclusion:**

The online multimodal psychological support program effectively enhanced the psychological well-being and sleep quality of new ICU staff demonstrating the potential of off line training in managing stress and improving health outcomes during crises. The findings of this study emphasize the importance of accessible, flexible psychological support, especially in high-stress environments such as ICUs during pandemics.

## 1 Introduction

Despite the adoption of stringent measures to contain the spread of COVID-19 in China (1), the healthcare system encountered a sudden increase in COVID-19 cases and ICU admissions following the relaxation of these restrictions in late 2022 (2–4). Owing to the shortage of ICU staff, physicians and nurses from other specialties underwent brief training and worked as part of the ICU care team (5). This experience, along with frequent work shifts and strict quarantine requirements, exacerbated stress in new ICU staff (6–9).

Several healthcare institutions are currently adopting support initiatives, advocating for self-care practices, and endorsing mental health resources to assist medical professionals in managing stress (10, 11). Many of these support resources facilitate open communication; provide access to counseling services; and foster a more supportive work environment inclusive of music, lectures, professional assistance, relaxation techniques, meditation, and other interventions targeting chronic stress (12). Although these in-person programs represent an important step toward improving the physical and mental well-being of healthcare professionals, they can be costly to organizations and healthcare professionals may experience difficulty in accessing the resources (13). Moreover, public health crises can increase the requirement while decreasing access to in-person psychological support resources, as evident by the social restrictions imposed during the COVID-19 pandemic (14).

To overcome the limitations of in-person interventions, we propose the use of online psychological support programs to reduce and manage stress among healthcare professionals as well as the general population (15, 16). Although internet-based psychosocial support programs have shown some benefits (17), the acceptability and effectiveness of programs that integrate diverse treatment modalities remain unclear (18). Moreover, these programs have not been systematically evaluated in the context of a global pandemic, wherein healthcare professionals are faced with sudden transitions in their work environment and responsibilities (19). We hypothesized that the online multimodal psychological support (MPS) program might improve the psychological well-being and sleep quality of participants immediately after the end of the intervention (which was 28 days after the participants were assigned to the ICU) at the 1-month follow-up (which was 28 days after the end of the intervention).

## 2 Methods

### 2.1 Participants

Healthcare professionals specializing in non-ICU fields such as anesthesiology, medicine, and surgery, including doctors and nurses, were recruited from the Second Affiliated Hospital of Zhejiang University School of Medicine, a quaternary-level hospital designated for the care of patients with COVID-19 in the Zhejiang province, from January to April 2023. These professionals had previously played an active role in caring for critically ill patients with COVID-19 during the beginning of the pandemic. Healthcare workers who were actively involved in the care of patients with COVID-19 and provided written informed consent were included. However, healthcare workers who declined participation or were unable to provide written informed consent were excluded. Moreover, healthcare workers who received prior relevant counseling or had severe mental illnesses were also excluded.

### 2.2 Procedure

This single-center, single-blind, randomized controlled trial was conducted at the Second Affiliated Hospital of Zhejiang University School of Medicine. This study was approved by the Institutional Review Board (Ethics Committee) of the Second Affiliated Hospital of Zhejiang University School of Medicine (20230401) and the National Institutes of Health Clinical Trials (approval number: NCT05713305, registration date: 01/18/2023). The included participants provided written informed consent and completed a pre-intervention (baseline, T0) psychological questionnaire within 2 weeks of their assignment to the ICU. Demographic information, including age, sex, marital status, family factors, occupation, job title, and personal or family history of psychiatric illness, was self-reported at baseline. Subsequently, the project coordinator randomly assigned participants to the MPS (online psychological support with multiple intervention modules) and RWC (routine health support) groups in a ratio of 1:1 using a computerized random number generator, with stratification by gender and occupation. The coordinator was not involved in intervention management or evaluation.

After 28 days of baseline assessment, the participants were asked to respond to a second questionnaire (T1) that was similar to the initial questionnaire. Approximately 2 months after baseline assessment, the participants were asked to respond to a third questionnaire (T2), which was also similar to the initial questionnaire. The longitudinal design allowed for the assessment of changes over time within each individual, controlling for their baseline level of distress. The participants received $50 as compensation for completing all assessments. Research assistants, blinded to the conditions of the participants, distributed the questionnaires either via E-mail or in person. The trial was concluded with the study end date and cessation of funding. Surveys are submitted anonymously online and participants are encouraged to complete the survey in a quiet environment.

### 2.3 Intervention (MPS group)

The MPS program was designed by a team of clinical psychologists and physicians based on in-depth interviews with 11 healthcare workers who sought psychological support during the COVID-19 pandemic (Supplementary Table S1). It was specifically designed to prevent and alleviate the most common psychiatric problems (depression, anxiety, stress, and insomnia) among healthcare workers dealing with the COVID-19 pandemic. WeChat was used to implement multimodal self-help intervention that included written and audiovisual content targeting four areas: music, sleep education, mental health education, and relaxation training. In addition, the intervention included daily reminders (notifications), which involved short questionnaires for providing feedback on the intervention (Supplementary Figures S1–S3), followed by the provision of information and resources tailored to the responses of individual participants. Examples include prolonging music sessions, increasing the content of relaxation sessions, and repeating sleep-assisting skills. Adjustments remain within the predefined framework. The intervention is described in detail below and in Supplementary Table S2.

During the time of implementation of the intervention, participants were offered and reminded to study or practice at a regular time each day through their own choices. We also keep statistics on the frequency and duration of the participants’ participation in training or learning, as well as their preference for self-selection. Psychological services were made accessible to both the MPS and RWC groups. All participants were encouraged to consult a psychologist if their symptoms deteriorated.

#### 2.3.1 Music

A clinical psychologist in our research team oversaw implementation of music therapy. Music therapy encompassed activities such as listening to pre-recorded music (20, 21); participating in online instrumental performances; and integrating music with other therapeutic modalities such as movement, imagery, and art (22).

#### 2.3.2 Sleep hygiene education

Participants were educated on health-related practices and environmental factors promoting sleep, such as light management, white noise utilization, temperature regulation, and the importance of including sleep-inducing music (23, 24).

#### 2.3.3 Psychoeducation

The COVID-19 Universal Mental Health Handbook serves as the principal source of information for psychoeducation (25, 26). The manual offers guidelines on the prevention and treatment of psychological disorders associated with COVID-19 and strategies for managing burnout, alleviating anxiety and depression, and fostering a healthy lifestyle (27, 28).

#### 2.3.4 Relaxation training

Anxiety levels can be effectively decreased using relaxation imagery and muscle relaxation techniques (29, 30). In this study, relaxation training included muscle relaxation, breathing exercises, and meditation. The participants could conveniently attend the training sessions online, specifically within online chat communities.

### 2.4 Routine health support (control, RWC group)

Participants in the RWC group were provided access to the COVID-19 Universal Mental Health Handbook (similar to the MPS group) via the WeChat platform.

### 2.5 Outcome measures

The primary outcome was overall psychological well-being, which was measured using the 21-item Depression, Anxiety and Stress Scale (DASS-21). This instrument has been widely used to assess psychological well-being among healthcare professionals, has strong internal consistency, and has been empirically validated in different populations, including healthcare professionals from diverse ethnic backgrounds (9). Multiple studies from China have investigated the psychometric properties of the DASS-21 instrument, revealing commendable internal consistency indices for depression, anxiety, and stress (Cronbach’s α values of 0.83, 0.80, and 0.82, respectively) (31–33).

Secondary outcomes included depression, anxiety, stress, and insomnia. Depression, anxiety, and stress were measured using DASS-21 subscale scores. Insomnia, both nighttime and daytime, was assessed using the Insomnia Severity Index (ISI), a seven-item Likert scale. The total score, ranging from 0 to 28, was calculated to assess the presence of insomnia within the study population (34).

### 2.6 Statistical analysis

To ensure a statistical power of 90% and a one-sided significance level of 5%, the sample size required to detect a minimum difference of 10 (±15) points in DASS-21 between groups was estimated to be 110 participants (with 55 participants in each group, accounting for a 10% attrition rate) (35, 36). The main population for analysis was defined as the modified intention-to-treat cohort, which included all patients who underwent any form of psychological intervention and completed a follow-up assessment of their mental health scores. A linear mixed-effect model was used to analyze changes in primary and secondary outcome measures before ICU assignment and 4 weeks after the end of 1-month ICU assignment in the MPS and RWC groups. Post-hoc analysis was conducted to compare the estimated marginal means at different time points within each group and between the two groups.

Sensitivity analysis was performed using a mixed-effect model, which included the baseline variables as confounders (Supplementary Table S3). Post-hoc analysis (as mentioned above) was performed to compare the estimated marginal means in the adjusted model. The *p*-value was determined using the multivariate t method, and results were averaged over the levels of all confounders from the linear mixed-effect model. Statistical analysis was performed using the R (version 4.2.2) software. The lme4 R package was used to establish the linear mixed-effect model, whereas the emmeans R package was used to calculate the estimated marginal means.

## 3 Results

**Figure 1:**
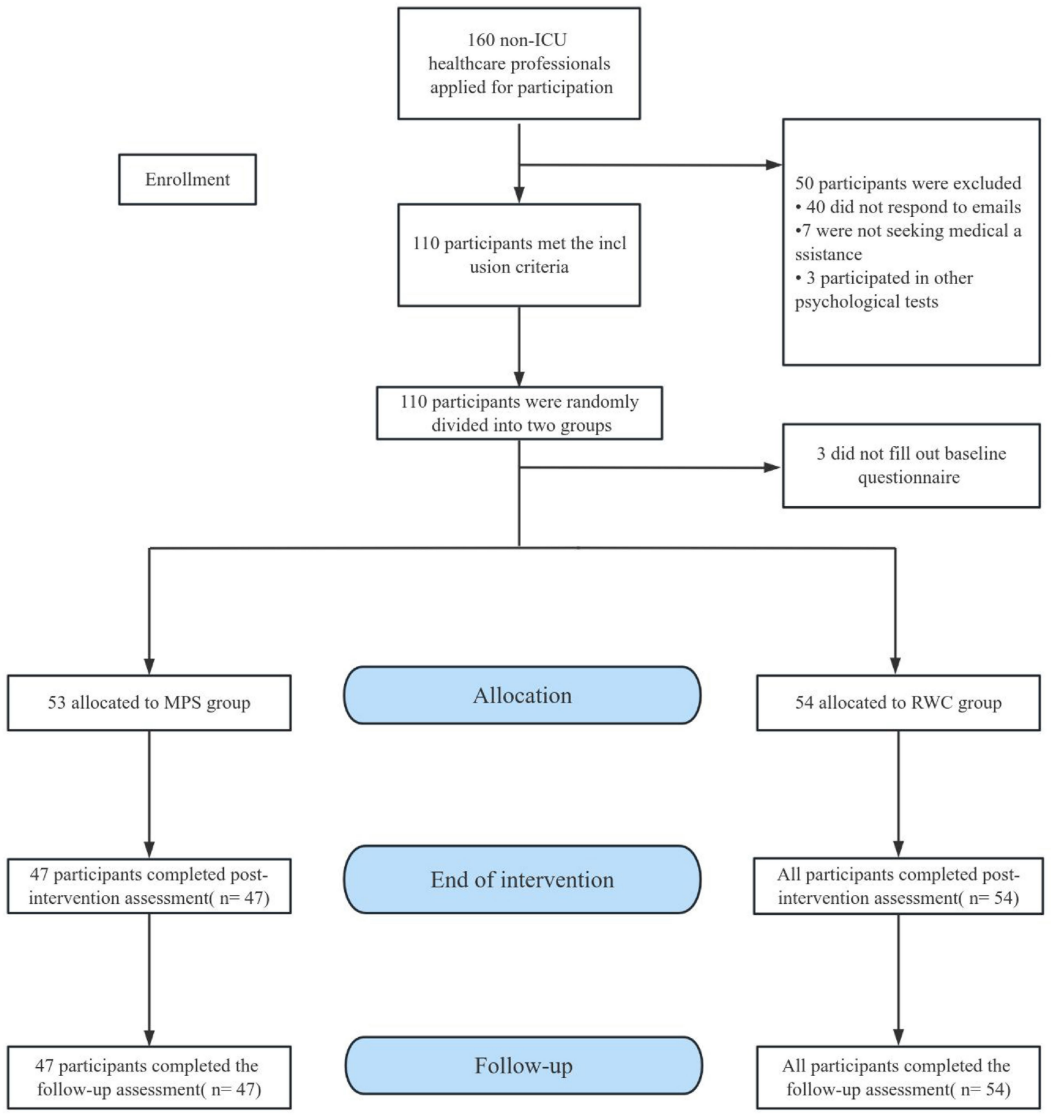
Flow chart. RWC, routine wellness care; MPS, multimodal psychological support.

A total of 160 eligible participants were screened from January 2023 to April 2023. Based on the inclusion–exclusion criteria, 110 participants were selected, with 55 participants being assigned to the MPS and RWC groups each. Subsequently, 3 participants were excluded because they did not respond to the baseline questionnaire, resulting in 53 and 54 participants in the MPS and RWC groups, respectively. At the end of the intervention, 47 of the 53 participants in the MPS group had completed the intervention and assessment. Therefore, a total of 101 participants were eventually included in data analysis, with 47 and 54 participants in the MPS and RWC groups, respectively (Figure 1). Among the participants, 63.0% were women and 37.0% were men, with 60.3% being physicians and 39.6% being nurses. The median age of the participants was 30.0 years, with an interquartile range (IQR) of 28.3–32.0 years, and the mean DASS-21 score at baseline was 33. The baseline characteristics of the MPS and RWC groups were comparable. The median DASS-21 score at baseline was 34 (range: 26–46) in the MPS group and 26 (range: 18–42) in the RWC group. Regarding mental health, symptoms of depression, anxiety, stress, and insomnia were observed in 53.4, 65.3, 37.6, and 65.3% of participants, respectively (Table 1).

**Table 1:** Baseline demographic characteristics of participants.

Characteristic	Intervention		*p* ^b^
	RWC, *N* = 54^a^	MPS, *N* = 47^a^	
Age	30.0 (28.3, 32.0)	30.0 (28.0, 34.5)	0.682
Sex			0.928
Male	20 (37%)	17 (36%)	
Female	34 (63%)	30 (64%)	
Profession			0.802
Nurse	22 (41%)	18 (38%)	
Physician	32 (59%)	29 (62%)	
Seniority			0.342
Junior	39 (72%)	28 (60%)	
Intermediate	13 (24%)	18 (38%)	
Senior	2 (4%)	1 (2%)	
Marriage			0.243
Unmarried	36 (67%)	26 (55%)	
Married	18 (33%)	21 (45%)	
Children			0.078
Do not have child/children	42 (78%)	29 (62%)	
Has child/children	12 (22%)	18 (38%)	
COVID vaccination			>0.999
No	2 (4%)	2 (4%)	
Yes	52 (96%)	45 (96%)	
ICU course			0.553
Not received ICU Short Training	25 (46%)	19 (40%)	
Received ICU Short Training	29 (54%)	28 (60%)	
Antidepressant			0.702
Not taking antidepressant	51 (94%)	43 (91%)	
Taking antidepressant	3 (6%)	4 (9%)	
DASS-21 score	26 (18, 42)	34 (26, 46)	0.078
Depression score	9 (2, 14)	10 (5, 14)	0.266
Anxiety score	8 (4, 14)	12 (8, 15)	0.065
Stress score	12 (7, 16)	14 (10, 18)	0.171
Insomnia score	9.5 (5.0, 13.0)	10.0 (7.0, 14.0)	0.384

a *n* (%).

b Wilcoxon rank-sum test; Pearson’s chi-squared test; Fisher’s exact test; RWC, routine wellness care; MPS, multimodal psychological support.

After 4 weeks, 47 of 53 (88.7%) participants in the MPS group were using the online support program. None of the participants developed an adverse event. Primary and secondary outcome data were available for 101 of 107 participants (94.4%). Without accounting for baseline covariates, the mixed-effect model provided estimates reflecting the average impact of the intervention on each mental health indicator. All estimates were negative, indicating that the intervention led to improvements in overall mental health. However, differences in total DASS-21 scores and individual depression, anxiety, and stress scores did not reach statistical significance (*p*-value > 0.05 for all). Notably, differences in ISI scores were significant, with an estimate of −2.028 and a standard error of 1.00, corresponding to a *p*-value of 0.0440 (Table 2).

**Table 2:** Mixed-effect modeling for primary and secondary outcomes.

Follow-up (MPS and RWC groups)	Estimate	SE	*p*
DASS-21	−5.69	3.92	0.1472
Depression	−1.84	1.47	0.2123
Anxiety	−2.05	1.33	0.1239
Stress	−1.79	1.42	0.2075
ISI	−2.028	1.00	0.0440*

Mixed-effect model with no baseline covariates. RWC, routine wellness care; MPS, multimodal psychological support; ISI, insomnia severity index.

*The differences found were statistically significant.

**Figure 2:**
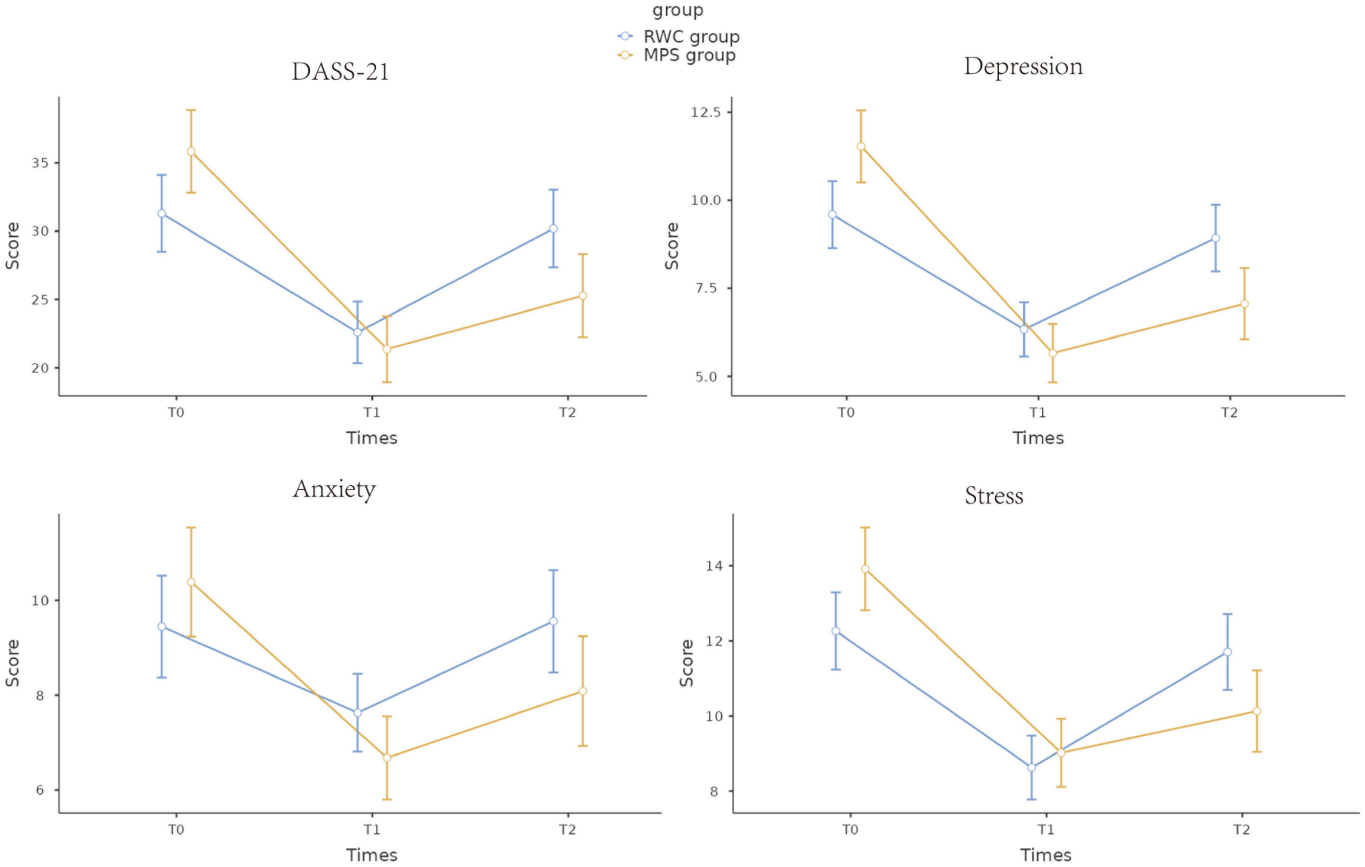
Changes in total DASS-21 scores and subscale scores during the intervention and follow-up periods. RWC, routine wellness care; MPS, multimodal psychological support; DASS-21, 21-item Depression, Anxiety and Stress Scale; T0, baseline; T1, immediately after the end of the intervention; T2, 4 weeks after the end of the intervention. DASS-21 and its subscale scores are expressed as the mean and standard error of the mean (SEM). The *p*-value represents statistically significant differences between the MPS and RWC groups at 4 weeks after the end of the intervention.

**Figure 3:**
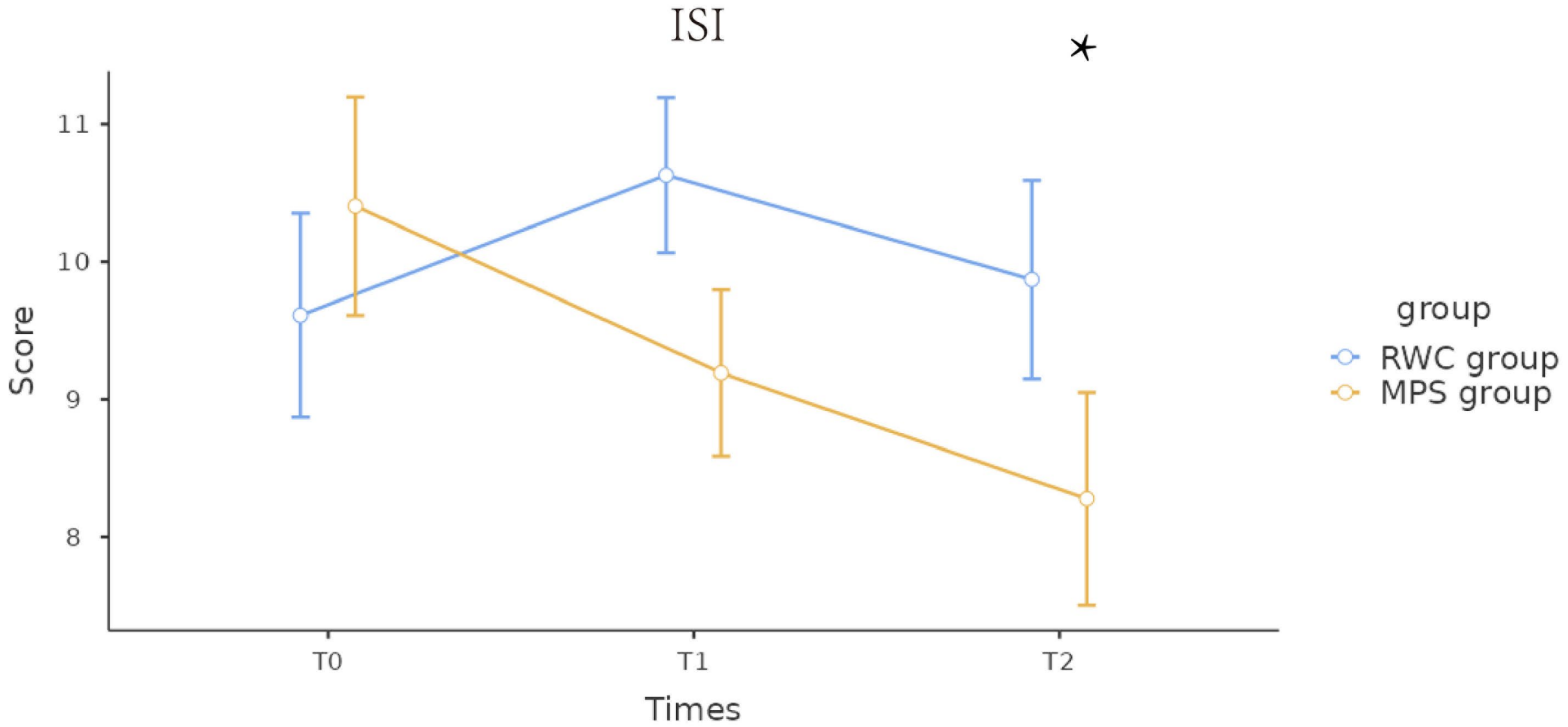
Changes in the Insomnia Severity Index scores during the intervention and follow-up periods. RWC, routine wellness care; MPS, multimodal psychological support; ISI, Insomnia Severity Index; T0, baseline; T1, immediately after the end of the intervention; T2, 4 weeks after the end of the intervention. Insomnia Severity Index scores are expressed as the mean and standard error of the mean (SEM). *p*-values represent statistically significant differences between the MPS and RWC groups at 4 weeks after the end of the intervention. * The differences found were statistically significant.

Furthermore, changes in mental health were assessed based on total DASS-21 scores and individual depression, anxiety, and stress scores at T0, T1, and T2. No significant differences in mental health indicators were observed between the MPS (intervention) and RWC (control) groups at T0. At the end of the intervention, the scores were lower in the MPS group than in the RWC group. The RWC group showed a reduction in stress and depression scores at T1 but a slight increase in anxiety, stress, and depression scores at T2 (Figure 2). Regarding insomnia, both groups had similar ISI scores at T0. The scores were not significantly different at T1 but were significantly lower in the MPS group than in the RWC group at T2 (Figure 3).

We counted the frequency and duration of training or learning attended by participants in the MPS group (Supplementary Figures S1, S2), as well as self-selection preferences (Supplementary Figure S3). And no participant seeking additional mental health counseling, with no significant side effects.

## 4 Discussion

This single-center randomized controlled trial demonstrated that Evidence-based online interventions have the potential to greatly benefit healthcare professionals working in ICUs. To our knowledge this is the first online intervention to include a real-time feedback adjustment system. In this study, we compared the effects of an online intervention program integrating music therapy, sleep hygiene education, psychoeducation, and relaxation techniques (MPS group) with those of standard health maintenance (RWC group) on healthcare professionals’ psychological well-being. Symptoms of anxiety, stress, and depression were significantly alleviated from baseline to the 1-month follow-up in both MPS and RWC groups. In addition, the MPS group showed a significant decrease in the severity of insomnia. Altogether, the online multimodal intervention program was feasible, without side effects, and was received well by the participants.

This study focused on a group that has often been overlooked in previous studies: new ICU staff who worked in ICUs during the COVID-19 pandemic and were responsible for taking care of critically ill patients (37). These individuals experienced a transition in their job responsibilities and work environment; however, the effects of this transition on their psychological state have not been investigated to date. This study emphasizes that prompt attention should be paid to the psychological problems of healthcare professionals working under tremendous pressure during global health crises (38, 39). The baseline data, satisfaction scores, and in-depth interviews in this study validated the mental health problems of healthcare professionals and the high acceptability of the online multimodal intervention program (40). Therefore, this study not only fills a knowledge gap but also provides a promising strategy for improving the mental health of healthcare professionals. We will conduct further research to develop more effective support and intervention strategies for improving healthcare professionals’ psychological well-being.

This study represents a novel addition to the existing literature, as it addresses the ongoing increase in the incidence of mental health disorders resulting from changes in the work environment and job responsibilities (41). During the COVID-19 pandemic, the healthcare system encountered considerable challenges owing to an increase in the number of critically ill patients. The online multimodal psychological support program used in this study was developed and executed by a multidisciplinary group of anesthesiologists, surgeons, and internists (42, 43). Designed to specifically address the challenges imposed by the COVID-19 pandemic, the MPS program incorporates evidence-based elements and can be tailored to meet individual requirements. Notably, the MPS program can be implemented via WeChat, a widely used communication platform in China, thereby offering flexibility and convenience under quarantined conditions during the pandemic (44).

This study is substantially different from previous studies. To date, most existing studies have used pre-determined content for participants to choose from; however, the MPS program developed in this study integrates components that have been shown to be effective. Therefore, this study introduces a novel strategy for adapting support content based on user feedback and the effectiveness of internet-based cognitive behavioral therapy in alleviating mental health disorders (45, 46). Compared with traditional wellness care, the MPS program resulted in a more significant reduction in the severity of psychological disorders at the end of 1-month ICU assignment among new ICU staff, which is consistent with the findings of previous studies, systematic reviews, and meta-analyses (38, 47).

Unlike existing single-modality interventions, the innovative MPS program represents a diversified and personalized approach to assessing the psychological well-being of healthcare professionals. Mental health interventions are crucial for alleviating emotional burnout and psychological burden among healthcare professionals (48). However, the effectiveness of single-modality interventions is often influenced by individual participant differences (49, 50). On the contrary, combining multiple interventions addresses a range of psychological issues and provides more comprehensive support (51, 52). This study showed similar findings, highlighting the importance of psychological interventions in the high-pressure ICU environment (53). The MPS program not only improved the sleep quality of the participants but also helped alleviate emotional stress, particularly leading to long-term improvements in the symptoms of anxiety and depression. Therefore, the online MPS program offers broad applicability and feasibility in emergency settings, providing a valuable basis for developing similar intervention strategies in the future.

The decrease in ISI scores in the MPS group provides a reference for investigating the effects of the MPS program on the sleep quality of healthcare professionals working in high-stress environments such as the ICU (48). This improvement in insomnia severity is especially noteworthy given the demanding nature of ICU responsibilities and their known impact on sleep quality. The trend toward “no clinically significant insomnia” in the MPS group highlights the efficacy of MPS in enhancing not only sleep quality but also overall well-being and job performance among healthcare professionals (49). On the contrary, the lack of changes in ISI scores in the RWC group emphasizes the requirement of targeted interventions, such as MPS, to address sleep disturbances among healthcare professionals working in high-stress environments.

We conducted an integrated analysis of participants’ frequency of engagement, total time invested, and preferences for different interventions to better understand their impact on DASS-21 scores (see Supplementary Figures S1–S3). The results indicate variability in both frequency and duration of engagement among participants, with those who engaged more frequently typically reporting improved mental health outcomes (50). This variability suggests that the extent of engagement may directly influence the efficacy of the interventions. Furthermore, the preference data shown in Supplementary Figure S3 demonstrates strong acceptance of relaxation techniques and music interventions, indicating that tailoring these interventions to individual preferences could significantly enhance their appeal and effectiveness (51).

### 4.1 Limitations

This study has certain limitations that should be acknowledged. This study was a single-center prospective randomized controlled trial that included new ICU staff working in ICUs during the COVID-19 pandemic. Therefore, the generalizability of the results may be limited. A double-blind approach was not practical in the context of the COVID-19 pandemic; therefore, demand characteristics and potential placebo effects cannot be completely ruled out when interpreting the results (52). However, the validity of the study is supported by the inclusion of exclusively new ICU staff from our center and the provision of a structured intervention through a pre-established online platform.

Future studies should test varying intervention dosage frequencies and durations and targeted content to determine their potential impact on depression, stress, and sleep (47, 53, 54). Additionally, given the overwhelming medical workload and the rapid spread of infection during the COVID-19 pandemic, weekly assessment of outcome measures over the intervention course was impractical in the present study. Future large-scale, multi-center randomized controlled trials should be conducted to assess the dose–response relationship with repeated longitudinal outcome measures and determine the feasibility, acceptability, and effectiveness of intervention strategies across medical contexts and healthcare professionals. No participants in this study used additional psychological counseling services, suggesting that future research should examine the protective effects of such counseling on outcomes.

### 4.2 Clinical implications

The novel, flexible online multimodal psychological support program developed in this study improved the overall psychological well-being of new ICU staff working in ICUs during the COVID-19 pandemic and could be easily implemented. This program holds potential for widespread application in clinical settings. In addition, as evidenced by its effects on insomnia, the multimodal psychological support program may help address the critical issue of sleep disturbance among healthcare professionals, thus improving their overall well-being.

## Data Availability

The raw data supporting the conclusions of this article will be made available by the authors, without undue reservation.
