# Supplementary material for: Enhancing the psychological well-being and sleep quality of healthcare providers with a multimodal psychological support program: a randomized controlled trial
Source: Front Public Health. 2024 Dec 24;12:1455174. doi: 10.3389/fpubh.2024.1455174 (PMC11703737; doi:10.3389/fpubh.2024.1455174)

Supplementary Table 1. Key Informant Interviews.

| Demand and Implementation |  |
| --- | --- |
| Working Factors | - Doctor A: During the initial weeks of ICU assignment, everyone was facing difficulties. The staff were cautious with their work. - Nurse A: As a new employee, I have no previous experience in dealing with this type of public health emergency, so it is difficult to relax no matter what I do. - Nurse B: The ICU environment is quite different from the ward environment. The constant alarm sound of the monitor makes me anxious, and I cannot communicate with the outside world with normal thinking and emotions. |
| Professional self-confidence | - Doctor B: On the one hand, the new colleagues are inexperienced; on the other hand, they lack a certain sense of responsibility and dedication in their work, which makes them very tired. - Doctor C: I really want to do something, but I do not know what I can do. I have been exposed to a completely unfamiliar professional field. I am constantly learning new skills every day and trying my best to do my job well. |
| Disease Outbreak | - Nurse C: The virus is too contagious, and my colleagues are constantly infected and unwell. I really want to quit my job and hide at home. - Nurse D: From the strict quarantine and the control of the pandemic in the beginning to complete liberalization now, many things have developed differently from my expectations. I have lost confidence in myself and experts and feel so powerless. |
| Emotional and Family Issues | - Doctor E: My wife and I just welcomed a baby. I was afraid I might infect my child. Therefore, I let my wife take our child back to my hometown. I go back to my empty home after work every day, missing my family. - Nurse F: My family understands and supports my work very much. After I fell ill, my parents contracted the infection as well, and I felt very guilty. |
| Emotional overreaction | - Nurse G: I always feel that my thinking is slower than usual and my words are not satisfactory. I cannot concentrate when I do things, but I pay too much attention to the real-time dynamics of the pandemic. - Doctor F: I am worried about going to work every day. There are too many critically ill patients in the ICU, and there is a possibility of needing rescue at any time. I feel that each day is like a year, and I am easily irritated, which leads to conflicts with colleagues because of trivial matters. |

Supplementary Table 2. Intervention module specifics and objectives

| Module | Theme | Tasks | Target |
| --- | --- | --- | --- |
| Module 1 | Music | - Soothing piano music from a professional psychologist - A lullaby to accompany you to sleep - Repeated practice and daily reminders to make it a habit | Anxiety, stress^22^, depression^34^, and insomnia^35^ |
| Module 2 | Sleep aid | - Popularization of sleep hygiene knowledge - Develop sleep habits with 28 days - Focus on the importance of sleep | Insomnia^24^, anxiety, and stress |
| Module 3 | Knowledge | - Learn to accept - Try to calm down - Calm and feel the warmth from the story - Constantly repeating and reviewing what you have learned before | Mental health^25^ and psychological adjustment needs^26^ |
| Module 4 | Lectures | - Scientific lectures on mental health - Identify positive emotions and be the master of your emotions - Identifying contemplation and worry | Mental health^36^, sleepiness, and quality of life^37^ |
| Module 5 | Relaxation | - Breathing exercises - Talking to yourself like a friend - Attention training - Develop an activity plan - Meditation practice - Muscle relaxation - Compassionate touch and stretch - Gratitude and embracing family members | Psychological symptoms, depression levels^27^, and quality of life |

Supplementary Table 3: Marginal means for primary and secondary outcomes

| Follow-up  (intervention and control groups) | Estimate | SE | P-value |
| --- | --- | --- | --- |
| DASS-21 | -5.62 | 3.95 | 0.1429 |
| Depression | -1.801 | 1.48 | 0.2261 |
| Anxiety | -1.991 | 1.34 | 0.1384 |
| Stress | -1.827 | 1.43 | 0.2040 |
| ISI | -2.041 | 1.02 | 0.0456* |

Mixed-effect model with no baseline covariates (intervention group, time + group * time, sex, age, profession, seniority, marital status, parental status, COVID-19 vaccination status, ICU training, antidepressant use)

Supplementary Figure 1: Number of interventions per participant in the MPS group


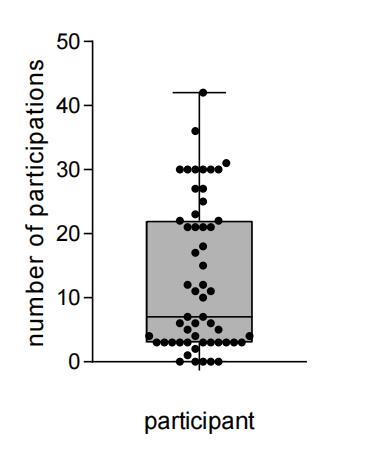


Supplementary Figure 2: Length of time each participant in the MPS group received the intervention


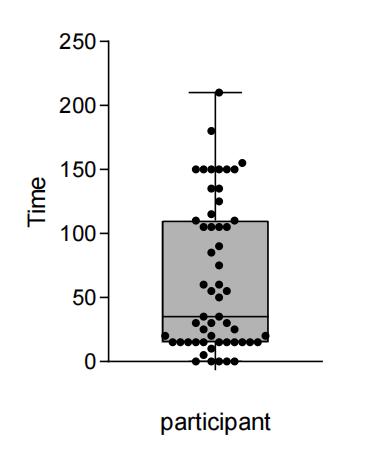


Time, min

Supplementary Figure 3: Preference of intervention content selected by each participant in the MPS group


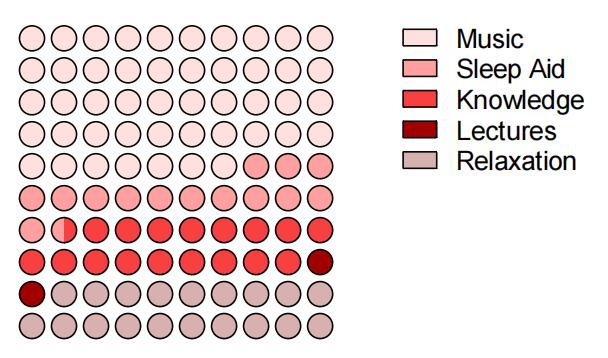

Supplement: Supplementary file 1 [file Table_1.docx]
